# Silencing of *Rac1* expression via RNA interference inhibits retinal neovascularization in rats

**Published:** 2012-05-30

**Authors:** Juanjuan Li, Yunpeng Li, Meixia Zhang, Zhulin Hu

**Affiliations:** 1 Department of Ophthalmology, Second People’s Hospital of Yunnan Province, Kunming, China; 2 School of Drug Prohibition, Yunnan Police Officer Academy, Kunming, Yunnan, China; 3 Department of Ophthalmology, West China Hospital of Sichuan University, Chengdu, Sichuan Province, China

## Abstract

**Purpose:**

To investigate the inhibitory effect of Ras-related C3 botulinum toxin substrate 1-small interfering RNA (Rac1-siRNA) on retinal neovascularization in a rat model.

**Methods:**

Rac1-short hairpin RNA (shRNA) was synthesized, constructed, and transfected into HeLa cells. Reverse transcription polymerase chain reaction was then conducted to test for *Rac1* gene expression. Retinal vein obstruction was performed in 25 Sprague-Dawley rats using the retinal photodynamic method. The vitrea bulbus of the eye in the shRNA rats group was transfected with the Rac1-shRNA vector, the other eye in the blank control group was transfected with the blank vector, and the interference control group was prepared by transfecting the Rac1-shRNA vector. Two weeks after transfection, the neonatal vessels were tested using fluorescein isothiocyanate–dextran retinal angiography. The number of endothelial cells beyond the internal limiting membrane was counted with hematoxylin and eosin staining.

**Results:**

A large area of neovascularization and fluorescein isothiocyanate leakage was found in the positive control group. However, a small area of neovascularization and a little fluorescence leakage were found in the shRNA group, whereas the retinal vessels were normal in the negative control interference group. In the shRNA interference group, the mean number of endothelial cells beyond the internal limiting membrane was significantly higher than that in the positive control group or the interference negative control group (p<0.05).

**Conclusions:**

Silencing *Rac1* expression with RNA interference inhibits retinal neovascularization in rats.

## Introduction

Neovascularization plays important roles in embryonic development and tissue injury restoration. Once the balance in neovascularization is disturbed, the normal physiologic state of organisms consequently changes. For example, inadequate neovascularization causes ischemia and unhealed ulcers, and excessive neovascularization causes tumors, immune system diseases, and vision loss. Among the diseases induced by unbalanced neovascularization, retina neonatal vascular ophthalmopathy accounts for the larger proportion. In ophthalmocace cases, including diabetic retinopathy, retinal vein obstruction, retinopathy of prematurity, and so on, the occurrence of neovascularization, as well as its induced pathologic changes such as hemorrhage, exudation, and hyperplasia, can disrupt ocular structure and function, which eventually causes severe visual impairment [1-3].

Retinal neovascularization occurs in retinal ischemia, hypoxia, or retinal circulation disorder. The new vessel inducer and inhibitor coregulate the formation of new vessels, and any disturbance in their balance, such as the number of inhibitors decreasing or the number of inducers increasing, can lead to neovascularization [4,5].

Vascular endothelial growth factor (VEGF), the most important of the different regulatory factors in neovascularization, can promote endothelial cell proliferation, intravascular component leakage, extracellular matrix change, and, ultimately, new vessel formation.

During neovascularization, Ras-related C3 botulinum toxin substrate 1 (Racl) regulates the expression and activity of hypoxia-inducible factor-1α (HIF-1α) [6,7].The *Rac1* gene is located at 7p22 in human cells, and is widely expressed in various tissues. The gene is a typical housekeeping gene [8]. During neovascularization, hypoxia leads to the phosphorylation and oxidation-reduction reaction of HIF-1α-related proteins. HIF-1α overexpression can increase the expression of downstream genes (such as VEGF, erythropoietin, glycolytic enzyme, etc.) as an adaptive adjustment of body tissues to hypoxia, which ultimately causes new vessel formation [9]. Rac1 can exert its inhibitory effect on neovascularization by inhibiting HIF-1α expression through several pathways [10,11].

Gene therapy has provided a broad research field for treating neovascularization diseases. The methods used in neovascularization include gene replacement therapy, antisense RNA therapy, and so on. RNA interference (RNAi) is a technology that uses small interfering RNA (siRNA) to specifically silence homologous gene expression, and has been widely used in treating ocular new vessels, ocular tumors, keratonosus, lens diseases, glaucoma, etc. [12-16].

Thus, silencing *Rac1* expression breaks the upstream signaling pathway of neovascularization, effectively inhibiting the formation of new vessels. Based on these findings, a Rac1-siRNA vector was constructed in the present study, and reverse transcription polymerase chain reaction (RT–PCR) was used to investigate the inhibitory effect of Rac1-siRNA on *Rac1* expression. Animal models of retinal neovascularization were established using the photodynamic method, and we observed the inhibitory effect of Rac1-siRNA on retinal neovascularization in a rat model.

## Methods

### Vector construction

The complete sequence of the human Rac1 mRNA was obtained from NCBI (GenBank AB029508). A total of 87 siRNA strands of human Rac1 mRNA at different loci of the coding region were designed by the siRNA software company (Ambion, Wizard, Dharmacon, Co. Ltd, San Antonio, TX). Three sequences were selected according to the following requirements: (a) the sequence was as close as possible to the designed primer region, and (b) the sequence was homologous with that of rat Rac1 mRNA. The homologous sequences between the selected siRNA sequence and other gene sequences were excluded. The three siRNA sequences were as follows: (A) 5′-AGA CGG AGC TGT AGG TAA A-3′, (B) 5′-ATG TCC GTG CAA AGT GGT A-3′, and (C) 5′-AAA GAC ACG ATC GAG AAA C-3′. The siRNA oligonucleotide fragments were annealed to form the target double-stranded DNA, followed by digestion with BglII and Hin d III restriction enzymes and ligation into linearized pSUPER plasmids (Oligoengine Co. Ltd., Los Angeles, CA) digested with the same enzymes. The plasmids were then transformed into competent *E. coli* DH5α and screened on containing plates to produce the recombinant pSUPER–Rac1–short hairpin RNA (shRNA) vector. The plasmids were extracted and identified with digestion with BglII and HindIII and sequencing. The resultant shRNA vectors were designated pSUPER–Rac1–shRNA1, pSUPER–Rac1–shRNA2, and pSUPER–Rac1–shRNA3.

### HeLa cell transfection

HeLa cells were purchased from the Shanghai Cell Bank (Shanghai, China). The cells were cultured in Dulbecco’s modified Eagle’s medium supplemented with 10% fetal calf serum at 37 °C with 5% CO_2_. pSUPER–Rac1–shRNA and the pSUPER plasmid were introduced into the HeLa cells according to the instructions for Lipofectamine 2000 (Invitrogen, Carlsbad, CA). A day before transfection, 0.5–2×10^5^ cells were inoculated into six-well plates. Transfection mixtures containing Solution A (5 μg of plasmid and 100 μl of Opti-MEM medium, Invitrogen, Los Angeles, CA) and Solution B (10 µl of Lipofectamine 2000 and 100 μl of Opti-MEM medium) were prepared in 96-well plates. After incubation for 4 h at 37 °C and 5% CO_2_, the transfection medium was replaced with complete medium, and the cells were incubated for 44 h at 37 °C and 5% CO_2_. After transfection for 48 h, the cells were removed from the plates, washed twice with precooled PBS (NaCl 8.00 g/l, KCl 0.20 g/l, Na_2_HPO_4_•12H_2_O 1.56 g/l, KH_2_PO_4_ 0.20 g/l; 1,320× g, 5 min), resuspended, and adjusted to 20×10^6^ cells/l.

### Reverse transcription polymerase chain reaction

The cells were collected from each group, and the total RNA was extracted using TRIzol reagent according to the kit’s instructions (TakaRa, Okinawa, Japan). The cells were reverse transcribed using the Super Script First-Strand System RT–PCR kit (TakaRa), and the *Rac1* gene was amplified with PCR. The reaction conditions were as follows: 30 s of denaturation at 94 °C, 30 s of annealing at 55 °C, 30 s of polymerization at 72 °C, for 35 cycles. A β-actin (*ACTB*) fragment was amplified under the same conditions as the intercontrol. The experiment was repeated three times.

### Animal models and grouping

This study was performed in strict accordance with the recommendations in the Guide for the Care and Use of Laboratory Animals of the National Institutes of Health. The animal use protocol has been reviewed and approved by the Institutional Animal Care and Use Committee (IACUC) of the Second People’s Hospital of Yunnan Province, China. A total of 50 healthy and mature Sprague-Dawley rats were provided by the West-China Centre of Medical Sciences, Sichuan University. The Rac1-shRNA recombinant vector (the interference group) was transfected into the vitrea bulbi of the eyes of 25 rats, and the blank vector (the positive control group) was transfected into the other eye. The other 25 rats were used as the interference negative control group. Retinal vein obstruction in the gene interference and positive control groups was induced using the photodynamic method. Mydriasis was induced in rats by intraperitoneal injection of tropicamide at 15 mg to 20 mg per kg bodyweight. After the rats were anesthetized with 1.5% pentobarbital, tail vein puncture was performed. About 2% rose bengal was injected into the rats at 40 mg/kg bodyweight. Fixed under a laser ophthalmoscope mediated by 90 D preset lenses, all major retinal veins around the optic disc were photocoagulated with a 532 nm laser. Within 4 DD to 1 DD away from the optic disc, distinctive black thrombi formed in the veins. The vein thicknesses were uneven, with tortuous and dilated distal parts. The peripheral retinas of some rats were interspersed with small flake-like hemorrhage. After the models were established, Rac1-siRNA recombinant vectors were transfected into the vitrea bulbi of the gene interference group, blank vectors into the positive control group, and Rac1-siRNA recombinant vectors into the negative control group.

### Retinal cell transfection

Rac1-siRNA vectors and blank vectors were mixed with Liposome 2000 under sterile conditions at a ratio of 1:1. Four rats were transfected with recombinant and blank vectors. After routine anesthesia, a microinjector was inserted into the back of the cornea, and 20 pmol/3 μl of the mixture was injected into each eye. Four days after injection, the rats were sacrificed, and their eyeballs were enucleated and stored at −25 °C for 30 min. Then, 6 μm-thick sagittal sections were cut parallel to the optic nerve. Sections with intact ocular structure were selected, and the transfections were observed under a fluorescence microscope.

### Fluorescein isothiocyanate–dextran retinal angiography

Fluorescein isothiocyanate–dextran was dissolved in sterile SPSS to a final concentration of 50 mg/ml. After routine anesthesia and fixation, the thoracic cavity was opened by cutting the sternum. About 1 ml of fluorescein isothiocyanate–dextran solution was injected into the left ventricle. Perfusion was considered successful if the mouth, nose, and external ears turned yellow. The eyeball was enucleated and fixed with 4% formaldehyde. Under an operation microscope, the eyeball wall was cut along the limbus cornea, and the lens and vitreous were removed. The sensory layer of the retina was separated from the pigment epithelium layer with an iris repository, and was stretched and observed under a fluorescence microscope.

### Pathological observation

The rats were sacrificed, and their eyeballs were enucleated; meanwhile, the orientations were marked. The eyeballs were fixed in 4% formaldehyde solution, routinely dehydrated, and then embedded in paraffin. Continuous 6 µm-thick sagittal sections were made parallel to the optic nerve. The number of endothelial cells beyond the internal limiting membrane was counted under a microscope.

### Statistical analysis

Data were analyzed using the SPSS 13.0, Beijing, China, software, and p<0.05 was considered significant.

## Results

### Rac1 expression in HeLa cells

The RT–PCR results show that the three vectors significantly inhibited *Rac1* expression in HeLa cells. The *Rac1* expression inhibitory rates of shRNA1, shRNA2, and shRNA3 were 7.05%, 9.40%, and 31.25%, respectively. The inhibitory effect of shRNA1 was the most significant, so it was selected for use in subsequent animal experiments.

### Effect of short hairpin RNA on angiogenesis of the retina

**Figure 1:**
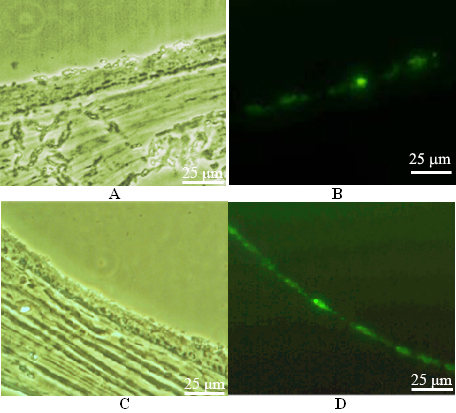
Cell transfection detected by fluorescence microscope. **A**: The image of the frozen section of rat eyeball wall in the control group under a microscope. Moderate green fluorescence was observed in the retinal region close to the vitrea bulbus 4 days after blank vector transfection. **B**: The image under a fluorescent microscope. Green fluorescence was seen in the retinal region. **C**: The image of the frozen section of rat eyeball wall in the gene interference group under a microscope. Moderate green fluorescence was seen in the retinal region close to the vitrea bulbus 4 days after recombinant vector transfection. **D**: The image under a fluorescent microscope. Green fluorescence was seen in the retinal region.

The selected recombinant shRNA vector and blank control vector was transfected into the rat’s vitreous cavity successfully, and moderate intensity fluorescence was observed (Figure 1). The constructed recombinant vector and the blank vector contained the cytomegalovirus (CMV) promoter that induced the expression of downstream green fluorescent protein (GFP) genes. The fluorescence proved the recombinant vector and the blank vector effectively induced gene expression in rat retinas.

The retina surface showed that the blank control group produced many new blood vessels, while the shRNA group had only fewer new blood vessels, and the blood vessels of blank control group was normal. The vascular endothelial cell numbers beyond the inner limiting membrane in the shRNA interference group were more than those of blank control and blank interference group, and showed statistical significance (p<0.05), which suggests that Rac1-siRNA could effectively inhibit the angiogenesis of the retina.

### Retinal angiography

**Figure 2:**
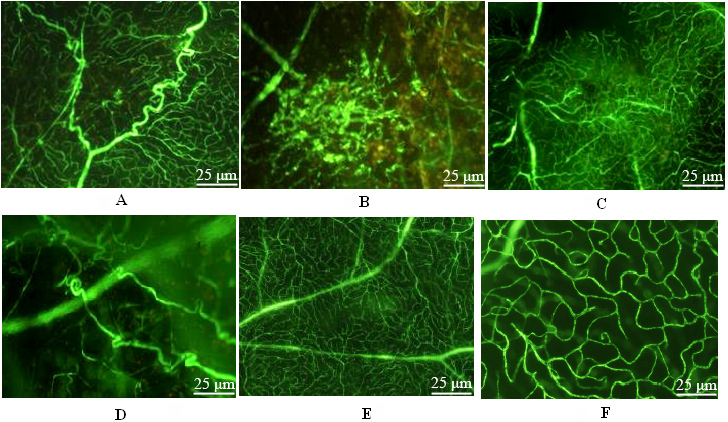
Retina preparation by fluorescein isothiocyanate-dextran heart perfusion. **A**: The image of rat retinal angiography in the positive control group under a fluorescent microscope. Tortuous and thick abnormal new vessels were seen. **B**: The image of rat retinal angiography in the positive control group under fluorescent microscope. Fluorescent leakage of new vessel mass was seen. **C**: The image of rat retinal angiography in the gene interference group under a fluorescent microscope. Vessels were distributed in a disorganized manner. **D**: The image of rat retinal angiography in the gene interference group under a fluorescent microscope. Tortuous and thick new vessels were found. **E**: The image of rat retinal angiography in the gene interference group under a fluorescent microscope. Two layers of retinal vessels in normal distribution. **F**: The image of rat retinal angiography in the gene interference group under a fluorescent microscope. Deep vessels were normally arranged as a net.

The retinal angiography of the positive control group showed that the retinal vessels had abnormal morphologies and distribution. Many abnormal neonatal vessel sprouts and neovascularization were observed, and fluorescent leakage exhibited strong fluorescence (Figure 2A,B). The shRNA group had abnormal retinal vessel morphology and distribution. New vessels and fluorescent leakage were observed but without a large area of neovascularization (Figure 2 C,D). The retinal vessel morphology in the negative control group was normal, and aberrant vessels or fluorescent leakage was not observed (Figure 2 E,F).

### Cells beyond the inner limiting membrane

**Figure 3:**
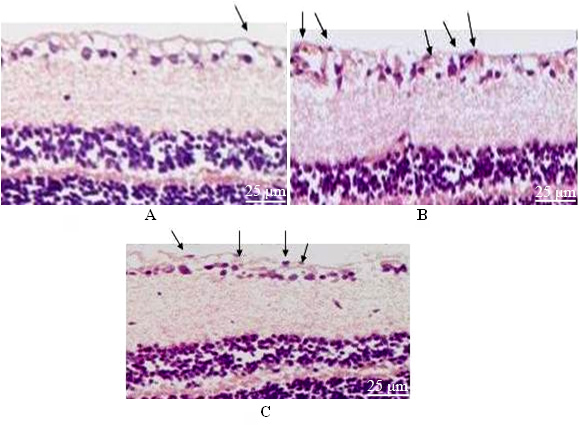
The nucleus number beyond retinal inner limiting membrane after HE staining. **A**: The image of the retina in the negative control group under a microscope. The endothelial cells were neatly arranged below the inner limiting membrane. **B**: The image of the retina in the positive control group under microscope. The endothelial cells broke through the inner limiting membrane and grew in a disorganized manner. **C**: The image of the retina in the short hairpin RNA group under a microscope. The number of endothelial cells increased, but only a minority of them broke through the inner limiting membrane.

The structures of the retinal sections from the three groups were clearly observed. Few vascular endothelial cells beyond the inner limiting membrane (Figure 3A) were observed, with a mean of 1.08±0.26 in the negative control group. The number of cells beyond the inner limiting membrane in the positive control group was higher, with a mean of 20.42 ± 2.36. However, the cells were arranged in a disorganized manner (Figure 3B). The number of vascular endothelial cells increased, but only a few cells beyond the inner limiting membrane were found, with a mean of 11.14±1.10 (Figure 3C). The differences among groups were statistically significant (*F*=47.168, p=0.000), and the differences in the number of cells beyond the inner limiting membrane between groups were statistically significant (*F*=23.768, p=0.002).

## Discussion

In the current study, we transfected the recombinant shRNA vector and the blank control vector into the rat vitreous cavity successfully, and moderate intensity fluorescence was observed. The retina surface showed that the blank control group produced many new blood vessels, while the shRNA group had only fewer new blood vessels, and the blood vessels of blank control group were normal. The vascular endothelial cell numbers beyond the inner limiting membrane in the shRNA interference group were more than those of blank control and the blank interference group, and showed statistical significance (p<0.05), which suggests that Rac1-siRNA could effectively inhibit angiogenesis of the retina. Some previous studies have proven that RNAi specifically and effectively inhibits VEGF expression, and inhibits the angiogenesis in a variety of disease models [17-20]. However, the application of RNAin in pertinent ophthalmological studies has not been reported.

In the present study, Liposome 2000 was used to mediate Rac1-siRNA transfection into the vitrea bulbus. Liposome mediation effectively increased the vector transfection effect, and transfection via injection into the vitrea bulbus resulted in localized high plasmid concentrations in the retina. Moderate fluorescence was observed in the retina in the frozen sections, which proved that the transfection was effective. We prepared the animal models of retinal neovascularization using the photodynamic method, and established different sample groups (shRNA interference, positive control, and negative control groups) to investigate the inhibitory effect of recombinant vectors on neovascularization and normal tissues. We found the retinal vessels in the shRNA group had abnormal structures and were arranged in a disorganized manner. New vessels and fluorescence leakage were also observed, but no neovascularization was found in the positive control group. Retinal sections were made to exclude interference from gliocytes, pericytes, and Müller cells. The number of vascular endothelial cells in the positive control group increased, and they were arranged in a disorganized manner. Many cells beyond the inner limiting membrane were found, whereas in the shRNA group, although the number of vascular endothelial cells increased, we observed only a few cells beyond the inner limiting membrane. The difference between these groups was statistically significant. Furthermore, the results demonstrated that the Rac1-siRNA recombinant vector had no obvious side effects on normal retinal tissues.

In summary, Rac1-siRNA has an inhibitory effect on multiple vascular growth factors and neovascularization in morphology and the expression of related growth factors.
